# Fumonisin production and symptom development in onion (*Allium cepa*) inoculated with *Fusarium proliferatum*

**DOI:** 10.1007/s12550-025-00595-0

**Published:** 2025-06-14

**Authors:** Sari Rämö, Sadikshya Ghimire, Minna Haapalainen, Satu Latvala

**Affiliations:** 1 Natural Resources, Natural Resources Institute Finland (Luke), Myllytie 1, 31600 Jokioinen, Finland; 2 Department of Agricultural Sciences, University of Helsinki, P. O. Box 27, 00014 Helsinki, Finland; 3 Natural Resources, Natural Resources Institute Finland (Luke), Tietotie 4, 31600 Jokioinen, Finland; 4 Department of Environmental and Biological Sciences, University of Eastern Finland, Yliopistokatu 7, 80101 Joensuu, Finland; 5 Natural Resources, Natural Resources Institute Finland (Luke), Latokartanonkaari 9, 00790 Helsinki, Finland

**Keywords:** *Fusarium proliferatum*, Inoculation, Onion, Gene expression, Pathogenesis, Fumonisin

## Abstract

*Fusarium proliferatum* is one of the main pathogens causing Fusarium basal rot of onion, which is a major problem in onion cultivation worldwide. In this work, three *Fusarium proliferatum* isolates from onion (*Allium cepa*), two from imported sets and one from a mature bulb grown in Finland, were tested for pathogenicity and mycotoxin production. Symptom development in the inoculated onion bulbs and production of fumonisins, beauvericin, and moniliformin were measured at 1–5 weeks post-inoculation (wpi). Symptoms were observed in all the bulbs inoculated with *F. proliferatum*, starting as water-soaked lesions and developing into dead brown scales. When colonizing the bulbs, all three *F. proliferatum* isolates were confirmed by qPCR to express the genes *FUM1*, required for fumonisin production, and *SIX2*, encoding a putative virulence factor. Fumonisin B_1_ (FB_1_) was detected already at 2 wpi in a symptomatic onion inoculated with Fpr047, and at 3 wpi, fumonisin production was confirmed for all three isolates. At 4 wpi, all the isolates had produced quantifiable amounts of FB_1_, ranging from 3.9 to 177 µg/kg, and fumonisin B_2_, ranging from 3.4 to 89 µg/kg. At 5 wpi, FB_1_ was even detected in a symptomless sample. Beauvericin and moniliformin were quantified at 5 wpi in the symptomatic onion tissues with Fpr047 and Fpr049, but not with FUS16163, which was unable to produce moniliformin and produced less beauvericin than the other *F. proliferatum* isolates also in the rice culture. The results showed that onions infected with *F. proliferatum* can contain high amounts of fumonisins.

**Supplementary Information:**

The online version contains supplementary material available at 10.1007/s12550-025-00595-0.

## Introduction

Fusarium basal rot (FBR) is a serious disease of onion (*Allium cepa*) and other *Allium* species worldwide, causing substantial losses both during the growing season and in storage after harvest (Le et al. 2021). In Finland, onions are produced either from imported sets or from pre-grown seedlings, due to the short growing season. The losses of onion yield caused by FBR have been estimated to be 30% in organic and 10% in conventional farming (Iivonen et al. 2014). In most cases, the primary pathogen causing FBR in onion has been identified as *Fusarium oxysporum* forma specialis *cepae*; however, *Fusarium proliferatum* (Matsushima) Nirenberg and *Fusarium solani* have also been frequently identified in the samples of onions with FBR symptoms in Finland and in many other countries worldwide (Toit et al. 2003; Ghanbarzadeh et al. 2014; Ravi et al. 2014; Haapalainen et al. 2016; Le et al. 2021). *F. proliferatum* was reported to be the predominant fungal pathogen found in the symptomatic bulbs of onion and garlic in Serbia (Stankovic et al. 2007) and in symptomatic garlic bulbs in Spain (Palmero et al. 2012; Gálvez et al. 2017) and Italy (Tonti et al. 2012). The *F. proliferatum* isolates from garlic were also found pathogenic on the seedlings of other *Allium* species, including leek (*A. ampeloprasum*), scallion (*A. fistulosum*), and chives (*A. schoenoprasum*) (Palmero et al. 2012).

*F. proliferatum* is a soil-borne pathogen that enters the onion plants through the roots (Carrieri et al. 2013). In our previous studies, all the *F. proliferatum* isolates from *Allium cepa* were pathogenic on both seedlings and mature bulbs and carried a putative virulence gene *SIX2*, variant *SIX2-1* (Haapalainen et al. 2023)*.* In addition, the majority (73%) of the *F. proliferatum* isolates had another variant *SIX2-2*, and 40% of the isolates carried one or two copies of another virulence-related gene *CRX2-FP* (Haapalainen et al. 2023), suggesting that specific molecular interactions between *F. proliferatum* and onion are required for the infection. Some *Allium* genotypes have been found to produce saponins that give the plant a partial protection against FBR; however, obtaining a high level of resistance has turned out to be challenging (Sharma et al. 2024).

Infection with *F. proliferatum* does not only spoil the quality of the onions but also causes a potential risk of mycotoxin contamination. The mycotoxin-producing ability of *Fusarium* isolates originating from onion, garlic, and asparagus has been studied in vitro by culturing the fungi on either maize or rice medium (Stankovic et al. 2007; Waśkiewicz et al. 2010; Irzykowska et al. 2012; Haapalainen et al. 2022). Fumonisin B_1_ (FB_1_), beauvericin (BEA), moniliformin (MON), fusaric acid, and fusaproliferin were identified as the main mycotoxin species produced by *F. proliferatum* isolates from onion and garlic when grown on maize kernels (Stankovic et al. 2007).

The main producers of fumonisins are *Fusarium verticillioides* (former *Fusarium moniliforme*) (Gelderblom et al. 1988) and *F. proliferatum* (Voss et al. 2007; Jurado et al. 2010). These *Fusarium* species spoil many important crops in tropical, subtropical, and temperate climate areas (Kang’ethe et al. 2017; Sun et al. 2019). The enzymes and regulatory proteins involved in the biosynthesis of fumonisins are encoded by the *FUM* gene cluster, and in *F*. *proliferatum*, the gene products *FUM1*, *FUM6*, *FUM8*, and *FUM21* were shown to be essential for the fumonisin production (Li et al. 2017; Sun et al. 2019). *FUM1* encodes the polyketide synthase required for the synthesis of fumonisin backbone (Proctor et al. 1999). For both *F. verticillioides* and *F. proliferatum*, the amount of *FUM1* transcripts showed a strong positive correlation with the amount of fumonisins produced in culture (López-Errasquín et al. 2007).

Of the different groups of fumonisins, B group is the most harmful and commonly recognized due to its toxic nature (Musser and Plattner 1997; Stępień et al. 2011). More than 60% of the total fumonisins produced by *F. proliferatum* is documented to be FB_1_, and the rest consists of fumonisin B_2_ (FB_2_) and fumonisin B_3_ (FB_3_) (Sun et al. 2019). The long-chain hydrocarbon unit of fumonisins has structural similarity to sphingosine and sphinganine and plays a role in their toxicity by disrupting the sphingolipid metabolism (Voss et al. 2007; Zain 2011).

FB_1_ is regarded as the most toxic within the B group, having adverse effects on the infected plants (Iqbal et al. 2021; Xie et al. 2021) and on humans and animals consuming the infected plant material (Stępień et al. 2011; Li et al. 2017). Feeding on moldy and FB_1_-contaminated maize has toxic effects on the liver, kidney, and cardiovascular system in horses and pigs (Voss et al. 2007). Similarly, multiple detrimental effects of fumonisins have been observed in chickens (Okasha et al. 2024). In humans, fumonisins are suspected to be a cause of esophageal cancer in many countries (Sydenham et al. 1990; Chu and Li 1994; Marasas 2001; Shephard et al. 2007; Kang’ethe et al. 2017). Fumonisin consumption has also been implicated in neural tube defects in infants (Zain 2011). In 2002, the evaluation by the IARC-International Agency for Research on Cancer (2002) stated that FB_1_ is possibly carcinogenic to humans (Group 2B), while in animal experiments, sufficient evidence for the carcinogenicity of FB_1_ has already been obtained.

By using ultra high-performance liquid chromatography-mass spectrometry (UHPLC-MS/MS) method, fumonisins, BEA, and MON could all be analyzed from the same samples of harvested onions, naturally infected with *F. oxysporum* and *F. proliferatum* and showing FBR symptoms (Rämö et al. 2021). In the onions infected with *F. oxysporum* alone, only BEA and MON were detected, whereas with mixed infection, with both *F. oxysporum* and *F. proliferatum*, fumonisins were detected in addition to BEA and MON. Similarly, in garlic cloves infected with both *F. proliferatum* and *F. oxysporum*, high concentrations of fumonisins FB_1_ and FB_2_ were detected (Mondani et al. 2021).

Since *F. proliferatum* was detected in onion in Finland and many fungal isolates were shown to be pathogenic to onion (Haapalainen et al. 2016, Haapalainen et al. 2023), it appeared important to study the mycotoxin production by these isolates in onion. The objectives of this study were to assess both the growth and mycotoxin production of *F. proliferatum* isolates within mature *A. cepa* bulbs, to monitor the development of visible symptoms and the expression of *F. proliferatum* genes associated with virulence and mycotoxin production, and to examine the relationship between fungal growth and mycotoxin production and the bulb symptom severity.

## Materials and methods

### Analysis of mycotoxin production in rice medium

Seven *Fusarium proliferatum* isolates, Fpr047, Fpr049, Fpr054, Fpr057, FUS15373, FUS16059, and FUS16163 (Haapalainen et al. 2016, Haapalainen et al. 2023; Table 1), were studied for their mycotoxin production capacity in rice medium. The *F. proliferatum* isolates had been stored cryo-preserved as part of the culture collection of Natural Resources Institute Finland and grown on potato dextrose agar (PDA) medium at room temperature (RT, 22 ± 1 °C) for 2 weeks prior to the rice culture experiment. Rice medium cultures were prepared similarly to those previously described for *F. oxysporum* (Haapalainen et al. 2022). Three replicate rice cultures were inoculated with each *F. proliferatum* isolate, and three subsamples were then extracted from each rice culture replicate. Mycotoxins were detected in rice culture samples as described in Haapalainen et al. (2022). The rice culture samples with mycotoxin concentrations over the highest calibration level, 500 µg/kg, were diluted and run again. An uninfected control rice extract was used as the diluent for the rice samples in ratios 1:9, 1:19, 1:99, 1:199, 1:499, and 1:999 (sample to diluent, v:v).

**Table 1:** Isolation source, genetic features, and pathogenicity on onion seedlings of seven *Fusarium proliferatum* isolates from onion (*Allium cepa*). The three isolates chosen for the onion inoculation experiment in this study are marked in bold

*F. proliferatum* isolate	Source of isolation*	Year of isolation	Pathogenicity on onion seedlings	GeneBank acc. no. for TEF sequence	Number of SIX-2 gene alleles detected	Reference
**Fpr047**	**Symptomless onion set**	**2013**	**Very pathogenic**	**Identical to KT239489**	**2**	Haapalainen et al. 2016; 2023
**Fpr049**	**Symptomless onion set**	**2013**	**Growth reduction**	**KT239488**	**1**	Haapalainen et al. 2016; 2023
Fpr054	Symptomless mature onion bulb	2013	Mild symptoms	na	na	Haapalainen et al. 2016
Fpr057	Diseased mature onion bulb	2013	Very pathogenic	KT239490	2	Haapalainen et al. 2016; 2023
FUS15373	Symptomless mature onion bulb	2016	Very pathogenic	Identical to OL763380	2	Haapalainen et al. 2023
FUS16059	Diseased mature onion bulb	2016	Pathogenic	OL763380	2	Haapalainen et al. 2023
**FUS16163**	**Symptomless mature onion bulb**	**2016**	**Pathogenic**	**OL763382**	**2**	Haapalainen et al. 2023

*na*, not applicable

*All the onion sets were imported to Finland, and all the mature bulbs were grown in Finland from imported sets

### Selection of *F. proliferatum *isolates for onion inoculation experiment

Based on the virulence to onion and genetic features (Haapalainen et al. 2016, Haapalainen et al. 2023; Table 1) and the mycotoxin production profile on rice medium (Table 2), three *F. proliferatum* isolates, Fpr047, Fpr049, and FUS16163, were chosen to be used for the onion inoculation experiment. Strong fumonisin producers Fpr047 and Fpr049 originated from symptomless onion sets (Haapalainen et al. 2016) and a weak fumonisin producer FUS16163 from a symptomless mature bulb (Haapalainen et al. 2023). Fpr047 had been shown to be highly pathogenic to onion seedlings, whereas Fpr049 only caused seedling growth reduction (Haapalainen et al. 2016). FUS16163 was pathogenic to both onion seedlings and mature onion bulbs (Haapalainen et al. 2023). The isolates Fpr047 and FUS16163 were found to carry two alleles of the putative virulence gene SIX2, whereas the isolate Fpr049 only harbored one allele (Haapalainen et al. 2023).

**Table 2:** Minimum (min) and maximum (max) concentrations (µg/kg) of fumonisins (B_1_, B_2_, and B_3_), beauvericin, and moniliformin in rice culture inoculated with *Fusarium proliferatum*. The isolates Fpr047, Fpr049, and FUS16163, used in the onion inoculation experiment in this study, are marked in bold

*F. proliferatum* isolate	Fumonisin B_1_ (µg/kg)		Fumonisin B_2_ (µg/kg)		Fumonisin B_3_ (µg/kg)		Beauvericin (µg/kg)		Moniliformin (µg/kg)	
	**min**	**max**	**min**	**max**	**min**	**max**	**min**	**max**	**min**	**max**
**Fpr047**	** < 10**	**69,000**	**1000**	**1900**	**4000**	**34,000**	**421,000**	**1267,000**	**200**	**600**
**Fpr049**	**13,400**	**24,600**	**2300**	**3300**	**3300**	**5300**	**756,000**	**962,000**	**700**	**1500**
Fpr054	1400	5600	200	1600	600	2200	105,000	137,000	< 2.5	200
Fpr057	1400	2200	500	700	500	700	< 2.5		nd	
FUS15373	1000	4600	1800	14,400	2800	10,000	595,000	961,000	50	105
FUS16059	300	1300	< 10	500	200	400	230	290	nd	nd
**FUS16163**	**100**	**300**	**70**	**130**	**90**	**110**	**50**	**110**	**nd**	**nd**

*nd*, not detected

### Onion material and inoculation

Yellow onion (*Allium cepa*) of the cultivar Setton harvested in autumn 2019 was used in this study. The onions had been grown organically from organic onion sets in Mikkeli in Eastern Finland. After harvest, the onions were dried at RT for a few weeks and stored for 3 months in a cold storage at 0.5 °C prior to the experiment. A hundred firm, healthy-looking, and similar-sized onion bulbs with no visible symptoms or mechanical injury were used for the experiment.

The *F. proliferatum* isolates (Fpr047, Fpr049, and FUS16163) were grown on PDA medium in the dark at RT for approximately 2 weeks before harvesting the spores for the inoculation experiment. Conidial suspension was prepared for each *F. proliferatum* isolate in sterile distilled water and filtered through sterile gauze, and the density was adjusted to 10^6^ conidia per mL. A 100 μL of inoculum suspension was injected into each onion bulb using a needle and a 1-mL syringe. The needle was pushed into the onion immediately adjacent to (not through) the basal plate, in an upward, angular direction towards the center of the onion to a depth of approximately 1–1.5 cm above the basal plate, as previously described (Haapalainen et al. 2022). The control bulbs were injected with sterile water. Twenty-five onions for each isolate or control were treated. All the treated bulbs were placed upside down on a cardboard support (Supplementary Data S1), so that they were not in contact with each other, and kept in the dark at 22 °C until sampling.

### Symptom area measurement

**Fig. 1:**
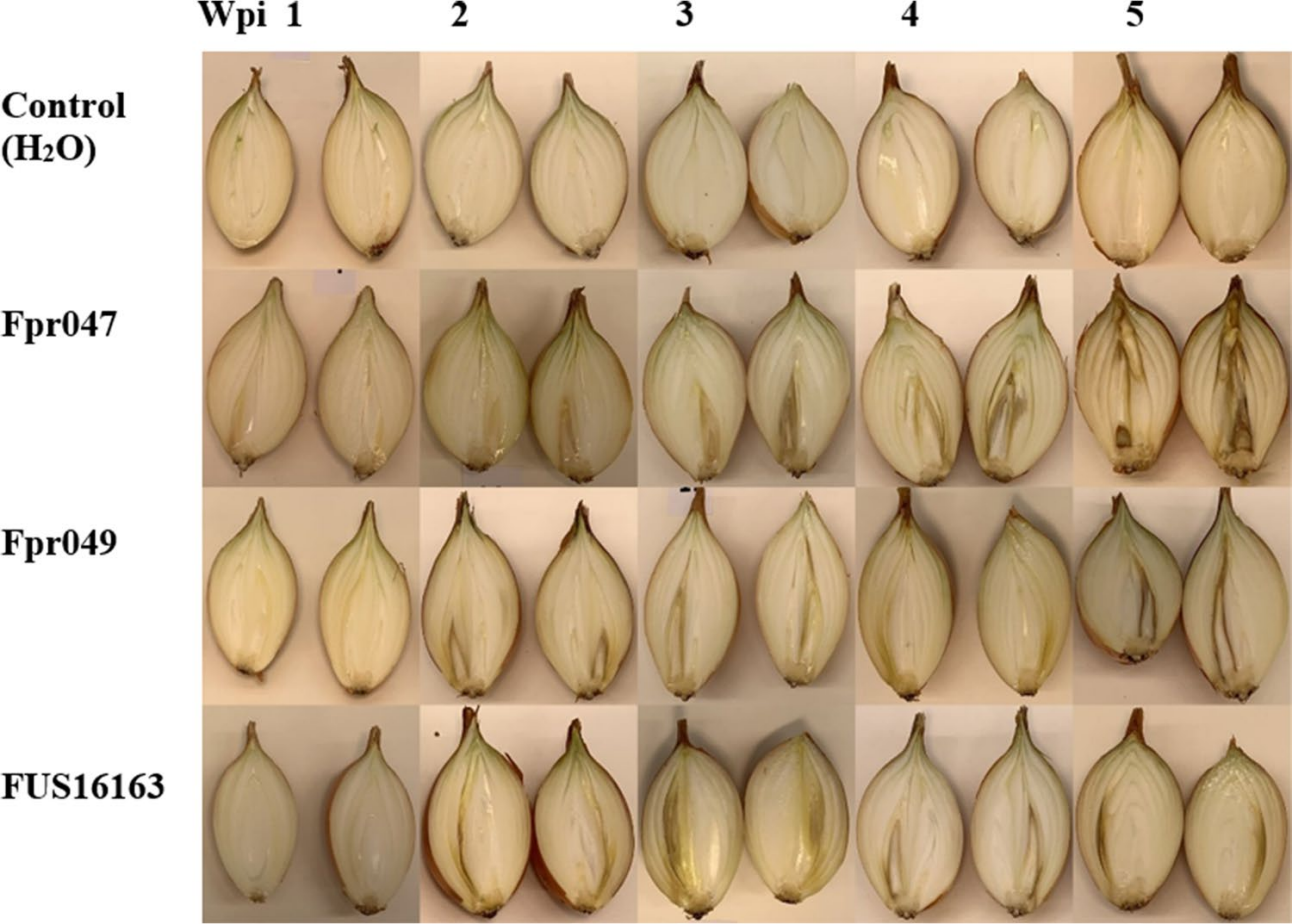
Symptoms observed in the onions inoculated with the three *Fusarium proliferatum* isolates Fpr047, Fpr049, and FUS16163, at 1 to 5 weeks post-inoculation (wpi). On the top are shown the water control bulbs at 1 to 5 wpi

A subset of bulbs from each inoculation treatment was sampled at five timepoints, from 1 to 5 weeks post-inoculation (wpi). At each timepoint, five bulbs per treatment were randomly picked as samples and cut into halves vertically from the point of inoculation, to observe possible symptoms of disease. The cut onions were photographed to record the symptoms (Fig. 1). These images were analyzed with ImageJ2 software (Rueden et al. 2017), by measuring both the symptomatic area and the whole area of the onion halves and then calculating the average proportion of symptomatic area for each bulb.

### Sampling for nucleic acid extraction and mycotoxin analysis

At each timepoint, three out of the five cut onions per treatment were taken for DNA and RNA sample preparation. Approximately 0.2 g of symptomatic (sample A) inner tissue and non-symptomatic (sample B) outer tissue was cut from each half of the onion, wrapped in aluminum foil, quickly frozen in liquid nitrogen, and stored at − 80 °C. For DNA and RNA extraction, the stored onion tissues were homogenized into fine powder with mortar and pestle in liquid nitrogen and stored in 2-mL microtubes at − 80 °C prior to extraction.

The remaining symptomatic (sample A) and non-symptomatic (sample B) onion tissues from the three cut onions per treatment were used for mycotoxin analysis. Three water-inoculated control onion samples (C) were also taken at each sampling point. These samples were stored at − 20 °C and then cut into small pieces with a sharp kitchen knife. For mycotoxin extraction, 1 g of a chopped sample was weighed in a 50-mL polypropene (PP) centrifuge tube with a screw cap. Three to five parallel subsamples were extracted immediately, and the rest of the samples were stored at − 20 °C.

### Preparation of nucleic acid samples

#### DNA extraction

DNA was extracted by the CTAB method, modified from Zhang et al. (1998). Each powdered onion sample was mixed in 800 μL of CTAB buffer (2% cetyl trimethyl ammonium bromide, 1.4 M NaCl, 20 mM EDTA, 0.2% 2-mercaptoethanol, and 2% PVP-40 in 100 mM Tris HCl, pH 8.0). The samples were incubated at 65 °C for 40 min, then extracted with 800 μL of chloroform octanol mixture (24:1), and centrifuged at 9400 × g for 5 min. The aqueous phase was collected, and DNA was precipitated with 800 μL of isopropanol at − 20 °C for 2 h, followed by centrifugation at 16,000 × g for 15 min at 4 °C. The DNA pellets were washed once with 70% ethanol and then dried and resuspended in 100 µL of nuclease-free water. DNA extraction from the *F. proliferatum* isolates grown as pure cultures on PDA was performed as previously described (Haapalainen et al. 2016). The DNA concentration was measured with a Nano-Drop Lite spectrophotometer (Thermo Fisher Scientific, Waltham, MA, USA), and samples were stored at − 20 °C for further use.

#### RNA extraction

Total RNA of the onion tissue samples was extracted using the Trizol method, according to Rio et al. (2010) with some modifications. Each powdered 0.1 g sample was dissolved in 1.5 mL of Trizol reagent (38% phenol (pH 4.3), 0.8 M guanidine thiocyanate, 0.4 M ammonium thiocyanate, and 0.1 M sodium acetate (pH 5.0), 5% glycerol), mixed by vortexing and incubated for 5 min at RT before centrifugation at 12,000 × g for 10 min. The aqueous phase was extracted with chloroform to remove the remaining phenol, and after centrifugation at 4 °C, RNA was precipitated from the aqueous phase with cold isopropanol for 10 min at RT, followed by centrifugation at 4 °C. The precipitates were washed with cold 75% ethanol, dried at RT, and dissolved in 70 µL of nuclease-free water for 10 min at 55 °C. To further purify the RNA, it was precipitated with 35 µL of 8 M LiCl at − 20 °C overnight. After centrifugation at 13,000 × g for 5 min at 4 °C, the pellets were washed with 350 µL of cold 75% ethanol and centrifuged again. The RNA was dissolved in 35 µL of nuclease-free water and stored at − 80 °C. RNA concentrations were measured with a NanoDrop Lite spectrophotometer.

To prepare control samples for studying plant-inducible gene expression, the *F. proliferatum* isolates Fpr047, Fpr049, and FUS16163 were grown as pure cultures on PDA medium. Fungal mycelia were scraped from the plates with a sterile loop and placed in a 2-mL safe seal tube and stored at − 80 °C, until homogenization and RNA extraction, following the protocol described above.

#### Synthesis of complementary DNA (cDNA)

To remove any contaminating genomic DNA from the RNA samples, aliquots of the RNA samples were treated with RQ1 RNase-free DNase I enzyme (Promega, Madison, WI, USA). The 10-µL reactions were prepared according to the manufacturer’s instructions, with 2 μg of the RNA sample. The obtained DNA-free RNA samples were used as templates for reverse transcription to produce complementary DNA (cDNA), by using RevertAid First Strand cDNA Synthesis Kit (Thermo Fisher Scientific) and random hexamer primers according to the manufacturer’s instructions (Thermo Fisher Scientific). For DNase control samples, 1 µL of water was added in each reaction instead of RevertAid M-MuLV Reverse Transcriptase. The prepared cDNA samples were diluted 1/5 in nuclease-free water before PCR amplification.

### Relative colonization level of *F. proliferatum* in onion

#### Preparation of DNA standard series

To determine the PCR efficiency and to quantify the fungal DNA concentration in the infected onion tissue samples, standard series were prepared. Serial dilutions were prepared of healthy onion DNA and *F. proliferatum* DNA at concentrations ranging from 0.03 to 27 ng/μL and 0.0002 to 0.2 ng/μL, respectively. The *F. proliferatum* DNA for preparing these standards was obtained from the isolates grown as pure cultures on PDA medium. After measuring the DNA concentrations by NanoDrop lite, a dilution series was prepared separately for each of the three *F. proliferatum* isolates—Fpr047, Fpr049, and FUS16163—with a stable onion DNA background, to mimic the samples prepared from the inoculated onions. The final dilution of the healthy onion DNA was 1/50, with a concentration of 16.27 ng/µL in all the *F. proliferatum* standards, equal to the sample dilution used when assaying the inoculated onions.

#### Quantitative PCR

In PCR, DNA extracted from healthy water-inoculated onions was used as the negative control, and DNA extracted from the isolate Fpr057 grown on PDA medium and mixed with healthy onion DNA was used as the *F. proliferatum* positive control. DNA samples from the inoculated onions were diluted 1/50 in nuclease-free water. Each 20 µL reaction contained SYBR Green I master mix (Roche, Mannheim, Germany) and either *F. proliferatum*-specific or onion-specific primers (Table 3) at 300 nM concentration and 5 μL of diluted DNA sample. The PCR was performed with Lightcycler 480 (Roche), using the following program: 95 °C for 5 min and 45 cycles of denaturation at 95 °C for 10 s, primer annealing at 60 °C for 10 s, and elongation at 72 °C for 10 s. For melting curve analysis, the program was as follows: denaturation at 95 °C and a temperature gradient from 65 to 97 °C. Three replicates of all the samples and standards were run, and Ct (cycle threshold) values were recorded.

**Table 3:** Primers specific to *Fusarium proliferatum* genes and intergenic spacer region and the onion (*Allium cepa*) reference gene used in PCR and qPCR in this study

Primer	Sequence 5′−3′	Target gene or region	Annealing temperature (°C)	Product size (bp)	Reference
FUM1-F1	GTGTATGGGGAGAGGATTGGC	FUM1	60.0	86	This study
FUM1-R1b	AAGTCATGCCCACCGGAGAC				This study
HistH3-F1	CCTTCCAGCGTCTGGTTCG	Histone 3	60.0	94	This study
HistH3-R1	TCGACGGACTCCTGGAGAG				This study
FprTEF-F2	TGTCACCGTCATTGACGCTC	Translation elongation factor 1α	60.0	116	This study
FprTEF-R2	CCTCGAACTCACCAGTACCG				This study
SIX2-1qF2	GTCCAATCTTTCGCTCAACAA	SIX2	60.0	99	This study
SIX2-1qR2	TCGGTGTAAATCTGCGTATCC				This study
FprIGS-F7w	GTGCAGACCAGAG**Y**GAACGTGGT	Intergenic spacer	60.0	90	Rämö et al. 2021
FprIGS-R7	CCCATCAGCCAGAGAACCGACATC				Rämö et al. 2021
28S-F	CGGTCCTGTAAGCAGTAGAGTAG	Intergenic spacer	64.0	535	This study
FIGS-R1	ACCTAGACCATACATCTCAACACG				This study
AcCOX1F (onion)	CGTGCTTACTTCACCGCAGCT	Cytochrome oxidase 1	60.0	163	Wang et al. 2019
AcCOX1R (onion)	TTCCTGTGAGCCCGCCTATGG				Wang et al. 2019

#### Calculation of the *F. proliferatum*/onion DNA ratio

The *F. proliferatum/*onion DNA ratio was calculated for each sample, based on the dilution series of *F. proliferatum* DNA mixed with healthy onion DNA, and the healthy onion DNA alone as a control. Standard curves were prepared by plotting the mean Ct values against the logarithm (log10) of DNA concentration. The equations of the standard curves, in the form of *y* = *a* + bx, were used to calculate the concentrations of *Fusarium* DNA and onion DNA in each sample. The logarithm of the concentration was calculated as *x* = (*y* − *a*)/*b*, where “*y*” is the average of the detected Ct values for each sample. The DNA ratio *Fusarium* vs onion was calculated for each sample, and the ratio was presented as *F. proliferatum*/onion (pg/µg).

### Determination of the relative gene expression levels

All the *F. proliferatum*-specific PCR primers (Table 3) were designed in this study. The primer design was based on the genomic assembly of *F. proliferatum* ET1 (Niehaus et al. 2016), Genbank GCA_900067095.1, except for the primer pair FprIGS-F7w/FprIGS-R7, which was based on a collection of intergenic spacer region sequences from several *F. proliferatum* isolates (Rämö et al. 2021). The program Primer3 (Untergasser et al. 2012) was used for designing the *SIX2*-specific primers. The *FUM1*-F1 primer was designed in two parts flanking intron 1, so that it would only give a product from the cDNA. Similarly, the HistH3-F1 primer was designed to span over the intron 2 junction of the *Histone 3* (*H3*) gene, and the FprTEF-F2 primer to span over the intron 3 junction, based on the gene sequences FPRO_13085 and FPRO_09277 of *F. proliferatum* ET1 (loci XM_031223672 and XM_031232653, respectively). The primer specificity was checked by real-time PCR on cDNA of additional RNA samples, before performing the analysis of the main samples. In total, 11 primer pairs were tested. PCR with the onion-specific primers (*COX1*) served as an internal positive control and was used for calculating the relative expression level of the *F. proliferatum* genes. To determine the reaction efficiencies of each primer pair, standards were prepared with a mixture of diluted (1/5) cDNAs. Serial dilutions were prepared of a combined cDNA sample with 1 µL of genomic DNA from Fpr047 (5 ng/µL) added in a total of 200 µL volume, to enhance the readings for the *SIX2-1* gene, which has no introns and can thus be tested with *F. proliferatum* genomic DNA. The PCR reaction volume was 20 µL, containing SYBR Green I master mix, 300 nM of the primers, and 5 µL of template cDNA. The real-time PCR reactions were run on the LightCycler 480 as described above.

For each primer pair, the PCR efficiency (*E*-value) was calculated as *E* = 10^(− 1/slope) from the obtained standard curves. Pfaffl model (Pfaffl 2001) was used for the calculation of relative gene expression levels of *FUM1* and *SIX2-1* in comparison to the reference genes *H3* and *TEF* and for calculating the mean cycle threshold (Ct) difference between the control and *F. proliferatum* samples. The expression levels of the gene of interest were normalized to the geometric mean of the reference gene expression levels, using the following equation:

1 $$\text{Gene expression ratio}=\frac{{E(GOI)}^{\Delta ct\left(GOI\right)}}{{E(ref)}^{\Delta ct(ref)}}$$

where E(GOI) and E(ref) are the PCR efficiencies for the gene of interest and the geometric mean of the reference genes, respectively, and Ct is the cycle threshold of the PCR. Ct was calculated as Δct (GOI) = control − treated (Ct of GOI), and Δct (ref) = control − treated (Ct of reference gene).

### Sequencing of the *F. proliferatum* intergenic spacer region

As the intergenic spacer (IGS) of the ribosomal RNA genes was used as the target sequence for species-specific detection and quantification of *F. proliferatum* in the onion tissue samples, the IGS of the three *F. proliferatum* isolates used in the inoculation experiment was sequenced. Amplification of DNA extracted from the isolates grown on PDA was performed by endpoint PCR with Phusion high-fidelity DNA polymerase (Thermo Fisher Scientific). The 50 µL reactions contained 1 × Phusion HF buffer, 200 µM dNTP mix, 500 nM of primers 28S-F and FIGS-R (Table 3), 0.5 µL of Phusion polymerase, and 3 µL of DNA template. The forward primer was modified from the 28S primer designed by Mirete et al. (2013). PCR was run on an Eppendorf MasterCycler Gradient thermocycler (Hamburger, Germany) using the following program: initial denaturation at 98 °C for 30 s, amplification for 40 cycles of 98 °C for 10 s, 64 °C for 30 s, and 72 °C for 30 s, final elongation at 72 °C for 10 min. The PCR products were analyzed by gel electrophoresis on a 1.0% agarose gel in Tris/acetic acid/EDTA buffer with ethidium bromide and visualized under UV light. The rest of the PCR products were purified by QIAquick PCR purification kit (Qiagen, Hilden, Germany) according to the manufacturer’s instructions. Sanger sequencing in both directions was performed at the sequencing laboratory of the Natural Resources Institute Finland. The forward and reverse DNA sequences were aligned using Clustal Omega (EMBL-EBI) and combined.

### Mycotoxin analysis of onion samples

Stock and standard solutions of FB_1_, FB_2_, FB_3_, BEA, and MON were prepared according to Rämö et al. (2021) for both rice medium and onion analysis.

Mycotoxins were analyzed in the inoculated and control onion tissue samples as described by Rämö et al. (2021) with minor modification: 1 mL of acetonitrile to water (1:1), instead of methanol, together with 50 µL of internal standard, was added to each 1-g onion sample. The calibration solvent of MycoMix solutions had to be replaced by acetonitrile to water (1:1) to achieve linear calibrations for fumonisins during revalidation of the method. Recovery tests were done in the non-symptomatic onion tissue used in Rämö et al. (2021) with four different concentrations: 1, 5, 10, and 100 µg/kg.

For each time point, at least three subsamples of each symptomatic and non-symptomatic tissue sample of the inoculated bulbs and three subsamples of control bulbs were analyzed. The onion samples with mycotoxin concentrations over the highest calibration level, 500 µg/kg, were diluted and run again. Mycotoxin-free onion extract was used as the diluent for the onion samples in ratios 1:1 and 1:9 (sample to diluent, v:v).

### Statistical analysis

All statistical analyses were performed in R studio, version 4.1.1 (2021–08–10). For the symptomatic area of onion bulbs inoculated with *F. proliferatum* isolates and the amount of the fungus in the onion tissue, normality of the data was tested using the Shapiro–Wilk test. Depending upon the normality of the data, a parametric or non-parametric test was utilized for pairwise comparison of the symptomatic area between different isolates at each time point. If all the groups (Fpr047, Fpr049, and FUS16163) at a given time point were normally distributed, a *t*-test was used. Whereas, if one of the groups at a given time point was not normally distributed, the non-parametric Wilcoxon rank sum test was used. The Wilcoxon signed rank test was used for comparison of the colonization levels between the symptomatic and non-symptomatic tissues in onions inoculated with the same isolate and to compare the different isolates to each other at each time point.

For analyzing correlation between the amount of *F. proliferatum* DNA and relative symptom area, between the *FUM1* gene expression level and relative symptom area, and between the *FUM1* gene expression level and measured concentrations of FB_1_ and FB_2_, the Pearson correlation test was used with a 95% confidence interval. For comparing the average expression levels of *FUM1* and the putative virulence gene *SIX2-1*, a two-sample *t*-test was performed (Supplementary Data S2), and samples of each *F. proliferatum* isolate were analyzed with one-way ANOVA.

## Results

### Mycotoxin production in rice medium

Fumonisins were produced by all seven *F. proliferatum* isolates studied, BEA was produced by six isolates, and MON by four isolates (Table 2). Because of high variation between the replicate samples, the results are given as minimum and maximum values. Based on the mycotoxin profiles in rice culture and other features discussed above in Materials and methods, the isolates Fpr047, Fpr049, and FUS16163 were selected for the onion inoculation experiment. In the rice culture, Fpr047 and Fpr049 were strong producers of fumonisin and BEA and also produced MON, whereas the isolate FUS16163 appeared as a weak BEA producer and did not produce MON.

### Symptom development in onion bulbs

The *F. proliferatum* inoculated bulbs looked healthy externally but showed symptoms in the internal tissues, whereas the control onion bulbs inoculated with sterile water remained symptom-free (Fig. 1). The symptom started as a slight brown area at the inoculated basal point and developed upwards into a V-shaped brown rot in the infected scales. At the time points 1, 2, and 3 wpi, the middle scales of the infected bulbs showed water-soaked lesions, which later turned dry, brownish to dark necrotic tissue.

**Fig. 2:**
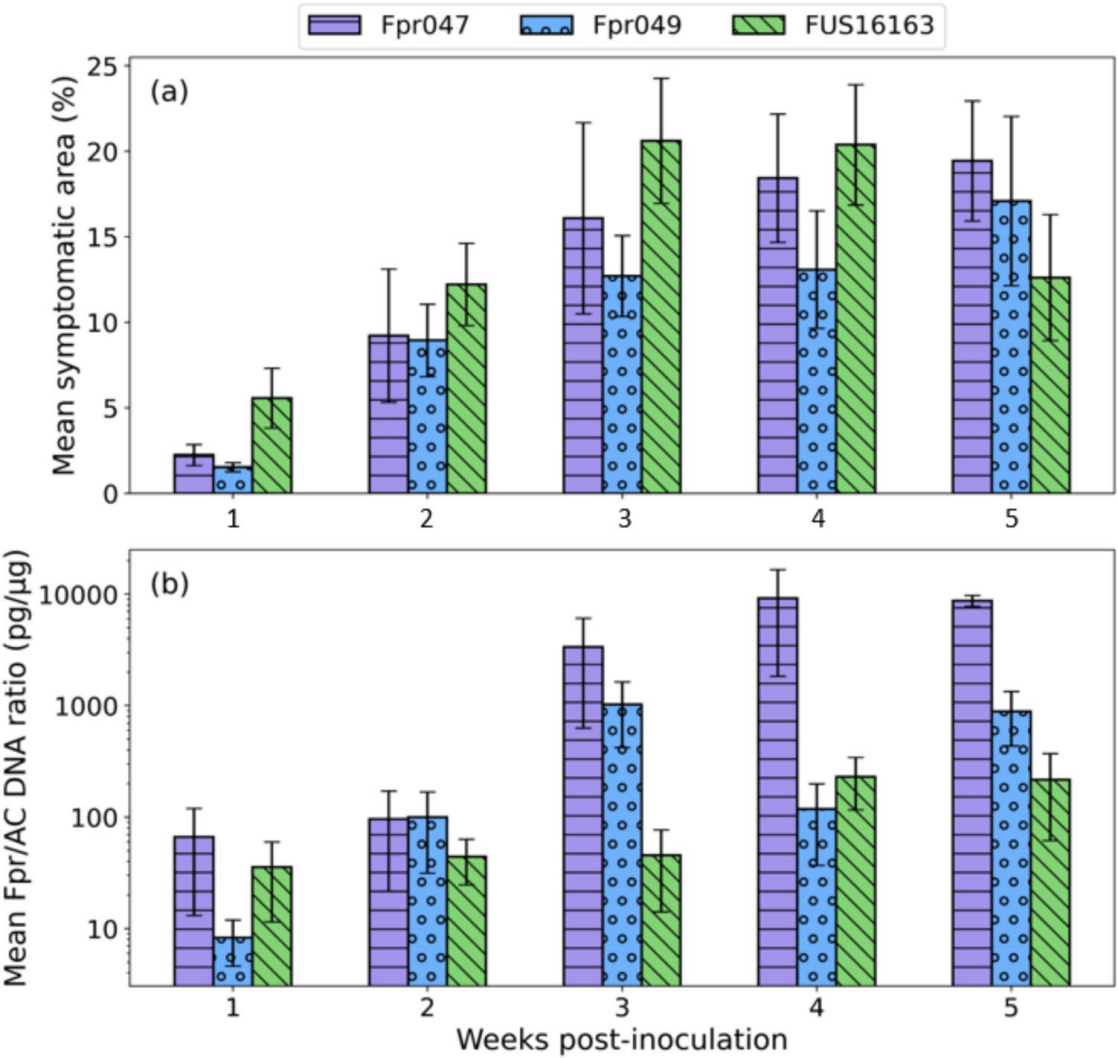
Mean symptomatic area (**a**) and *Fusarium proliferatum* (Fpr) colonization level in the symptomatic onion tissues (**b**) in the infected onion bulbs at different time points post-inoculation. The error bars show ± 1SE (*N* = 10)

Image analysis of the visual symptoms showed that with the isolate FUS16163, the percentage of symptomatic area was higher than with the other isolates across the time points 1–3 wpi. However, after 3 wpi, the increase in the symptomatic area ceased in the bulbs inoculated with FUS16163, while with the isolates Fpr047 and Fpr049, the symptomatic area still increased (Fig. 2a). For all the tested isolates, the observed maximal symptom area was more than three-fold as large as the symptomatic area at 1 wpi. The test of normality revealed that the data in all sample sets were normally distributed, except for Fpr047 at 2 wpi. Hence, the Wilcoxon test was utilized to analyze the difference of medians between the three isolates at 2 wpi, and for the other time points, a two-sample *t*-test was performed to analyze the significance of the difference of means. For all the isolates, the 1-wpi results were significantly (*p* < 0.05) different from the later time point results. For Fpr047, the median symptom area was also significantly (*p* < 0.01) higher at the time points 4 and 5 wpi in comparison to 2 wpi, and for FUS16163, the difference of the means was significant between 2 and 3 wpi (*p* < 0.05, *t*-test). For Fpr049, differences in means between the time points 2, 3, 4, and 5 wpi were not significant). When comparing the different isolates to each other at each time point, a statistically significant difference was only found between Fpr049 and FUS16163 at 3 wpi (*p* < 0.05, *t*-test).

### Colonization of the onion tissues with *F. proliferatum*

The amount of *F. proliferatum* DNA in the symptomatic and non-symptomatic tissues of the onions was quantified by qPCR at each time point. Unexpectedly, the qPCR with *F. proliferatum* IGS region primers (Rämö et al. 2021) had a lower detection sensitivity with the isolate FUS16163 than with the other isolates. To find out the reason for this, the IGS region of the three isolates used in the inoculation experiment was sequenced. Sequence comparison revealed variation between the isolates (GenBank accessions PQ352462, PQ352463, PQ352464). A total of 25 single nucleotide polymorphisms (SNPs) were found between the three isolates. FUS16163 sequence differed by 4.5% from the two other isolates, whereas Fpr047 and Fpr049 only differed from each other by 1.2%. The FUS16163 IGS sequence showed best matches (99%) to *F. proliferatum* strain NRRL_31914 (GenBank accession number MH398173.1) from winegrape and to the isolate GR_FP86 (MT340830.1) from asparagus root. Due to the divergence of FUS16163 from the other *F. proliferatum* isolates from onion, both the forward and reverse primer had a mismatch with FUS16163. In the forward primer, the mismatch of two nucleotides close to the 3′ end was likely to weaken the primer binding and thus reduce the PCR efficiency. However, as DNA standard curves were prepared separately for each isolate, the relative colonization levels could be calculated using these standard curves. The standard curve of FUS16163 had a higher intercept than the two other isolates (Eqs. 2–5).

2 $$Onion: y=-3.5495x+23.872$$

3 $$Fpr047: y=-3.6361x+19.671$$

4 $$Fpr049: y=-3.9472x+18.88$$

5 $$FUS16163: y=-3.5946x+23.115$$

At 4 and 5 wpi, the relative amount of *F. proliferatum* DNA in the symptomatic onion tissues was higher in the bulbs inoculated with the isolate Fpr047 than in the bulbs inoculated with the two other isolates (Fig. 2b). However, due to the large variation between the replicate samples, the difference between Fpr047 and the other two isolates was statistically significant only at 5 wpi. For Fpr047, the relative amount of *F. proliferatum* DNA was significantly higher in the symptomatic tissue in comparison to the symptomless tissue of the same bulbs (*p* < 0.01, paired Wilcoxon rank sum test), whereas for Fpr049 and FUS16163, a significant difference between the symptomatic and symptomless samples was not found. When all the symptomatic tissue samples inoculated with the same isolate were grouped together, the relative amount of *F. proliferatum* DNA was significantly higher in the bulbs inoculated with Fpr047 than in those inoculated with FUS16163 (*p* < 0.05, Wilcoxon rank sum test).

### Correlation between the symptom area and the relative amount of *F. proliferatum* DNA

Correlation between the symptom area, determined by image analysis, and the relative amount of *F. proliferatum* DNA, determined as base 2 logarithm of the Fpr/Ac DNA ratio, was analyzed. Three samples (Fpr047_2A, 1 wpi; Fpr047_19A, 4 wpi; Fpr049_24A, 5 wpi) were discarded as outliers, based on their qPCR results deviation from the overall dataset, and paired data for 42 samples were analyzed. The relative amount of *F. proliferatum* DNA and the average symptom area showed a significant positive correlation (Pearson: *r* = 0.43, *p* = 0.010).

### Relative gene expression levels

**Fig. 3:**
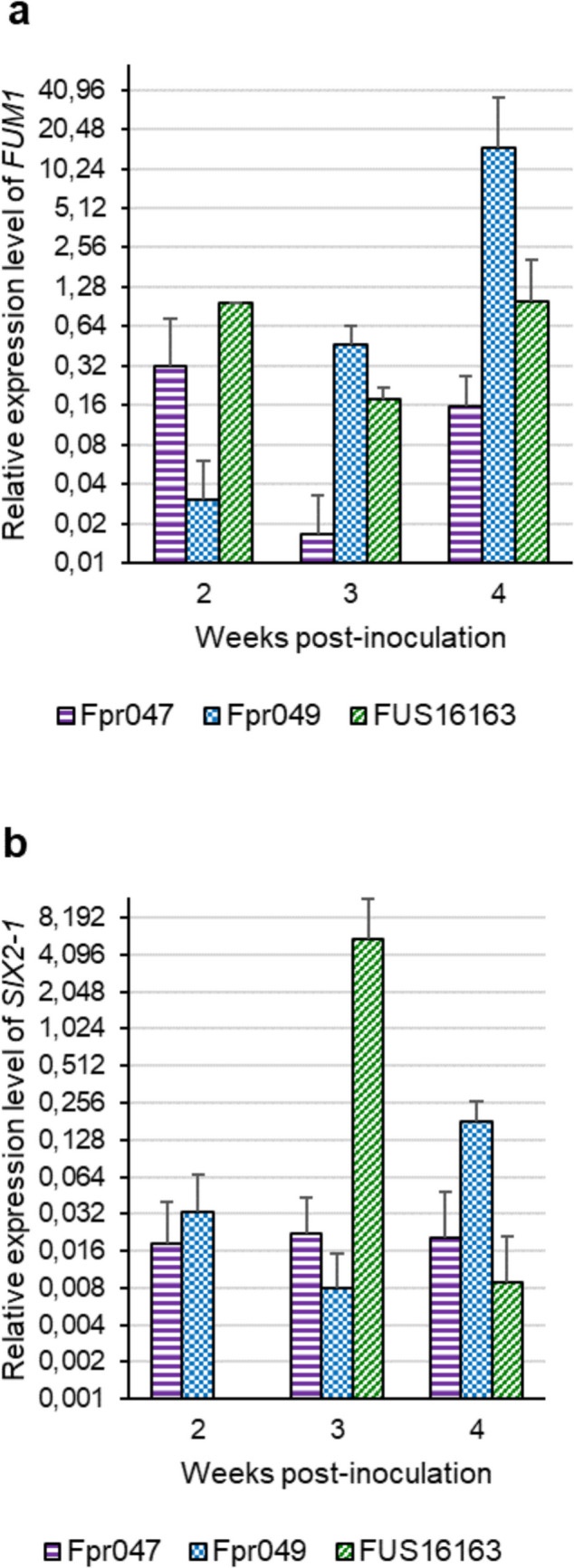
Average relative gene expression levels of **a** *FUM1* and **b** *SIX2-1* of the three *Fusarium proliferatum* isolates at different time points in samples of inoculated onions. The expression levels of *FUM1* and *SIX2-1* were normalized against the geometric mean of the expression levels of *F. proliferatum* reference genes *TEF1* and *H3*. The error bars represent standard deviation of the mean. Note that the *y*-axis scale is logarithmic

**Fig. 4:**
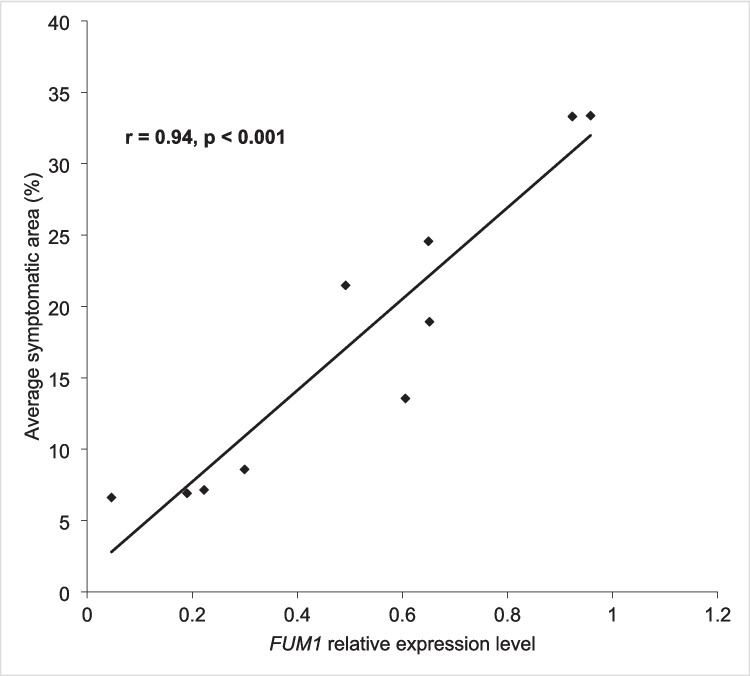
Correlation between the detected *Fusarium proliferatum FUM1* gene expression and average symptomatic area in the onion bulbs

Four primer pairs designed for *F. proliferatum FUM1*, *SIX2-1*, *H3*, and *TEF* performed well in PCR, with qPCR efficiencies 1.96, 2.05, 1.97, and 1.92, respectively, and with 1.98 for onion *COX1. F. proliferatum* gene expression levels were only measured from samples taken at 2, 3, and 4 wpi, as the fungal colonization levels were still low at 1 wpi and the symptom progression stopped by 5 wpi. In comparison to the isolate Fpr047, the isolates FUS16163 and Fpr049 had more samples expressing the *FUM1* gene, and at 3 and 4 wpi, the relative expression levels were also higher (Fig. 3a). The putative virulence gene *SIX2*-*1* was expressed at higher levels in Fpr047 and Fpr049 than in FUS16163 at 2 and 4 wpi, while in FUS16163, the expression level peaked at 3 wpi (Fig. 3b). The relative expression levels of *FUM1* were significantly higher than those of *SIX2-1* (Supplementary Data S2). The number of samples in which *SIX2-1* gene expression was detected was higher at 4 wpi than at 2 and 3 wpi, agreeing with the increasing amount of *F. proliferatum* in the infected onion samples. Significant differences were detected between the isolates in the expression levels of both *FUM1* and *SIX2-1*. At 2 wpi, there was a significant difference between Fpr049 and FUS16163 (*p* = 0.035, two sample *t*-test), and at 3 wpi, significant differences were found between all the isolates: Fpr047 and Fpr049 (*p* = 0.028), Fpr047 and FUS16163 (*p* = 0.025) and Fpr049 and FUS16163 (*p* = 0.012). For *SIX2-1*, a significant difference was only detected at 4 wpi between Fpr047 and Fpr049 (*p* = 0.035). The expression levels of *FUM1* and *SIX2-1* were low and several magnitudes below the expression levels of the reference genes *H3* and *TEF*. When the correlation between the symptom area and the *F. proliferatum FUM1* gene expression level was tested using the Pearson correlation test, a significant positive correlation was found (*r* = 0.55, *p* = *0.012*; Fig. 4).

### Revalidation results of the fumonisin quantification method in onion

The limit of quantification (LOQ) of fumonisins in onion was 2.5 µg/kg compared to earlier LOQ (10 µg/kg, Rämö et al. 2021) after revalidation of the method. The new limit of detection (LOD) was 0.5 µg/kg compared to earlier LOD (2.5 µg/kg, Rämö et al. 2021). The recoveries were 99 ± 3%, 100 ± 3%, and 103 ± 3% for FB_1_, FB_2_, and FB_3_, respectively, in quantitative area (2.5–500 µg/kg).

### Mycotoxin production in onion

The samples from water control onions were fumonisin-free at all five time points of sampling. In the *F. proliferatum*-inoculated bulbs, fumonisins were not detected in the samples of non-symptomatic onion tissues until 5 wpi, when FB_1_ was detected (< 2.5 µg/kg) in one sample inoculated with Fpr047. In the samples of symptomatic onion tissues, no fumonisins were detected in any of the samples at 1 wpi.

The first fumonisin positive sample was detected at 2 wpi: FB_1_ was quantified at 2.7 µg/kg in one subsample with Fpr047. However, the FB_1_ concentrations were still at trace level at 3 wpi in the samples with Fpr047 (Table 4). Although no FB_1_ was detected in the samples with Fpr049 or FUS16163 at 2 wpi, both FB_1_ and FB_2_ were at quantitative level in the samples taken at 3 wpi. The fumonisin concentrations showed a large variation between the samples and subsamples, and thus, the results are given as average, median, minimum, and maximum concentrations of symptomatic onion tissues since 3 wpi (Table 4). At 4 wpi, all three fumonisins were detected at quantitative level in bulbs inoculated with Fpr047 and FUS16163, but FB_3_ only at trace level in the samples with Fpr049 (Table 4). At 5 wpi, quantitative concentrations of FB_1_, FB_2_, and FB_3_ were detected for all three isolates (Table 4). The distribution of fumonisins inside the symptomatic area was more homogenous in the 5 wpi samples than in the 3 and 4 wpi samples.

**Table 4:** Fumonisins B_1_, B_2_, and B_3_ results given as average, median, minimum (min), and maximum (max) concentrations (µg/kg) in the subsamples of the symptomatic onion tissues inoculated with three *F. proliferatum* (Fpr) isolates at 3, 4, and 5 weeks post-inoculation (wpi)

Sampling point	Fpr- isolate	Fumonisin B_1_ (µg/kg)				Fumonisin B_2_ (µg/kg)				Fumonisin B_3_ (µg/kg)			
		**average**	**median**	**min**	**max**	**average**	**median**	**min**	**max**	**average**	**median**	**min**	**max**
3 wpi	Fpr047	< 2.5	nd	nd	< 2.5	nd	nd	nd	nd	nd	nd	nd	nd
	Fpr049	9	< 2.5	nd	63	4	< 2.5	nd	35	nd	nd	nd	nd
	FUS16163^a)^	3	nd	nd	26	< 2.5	nd	nd	26	nd	nd	nd	nd
4 wpi	Fpr047	15	6	nd	111	5	3	nd	35	< 2.5	nd	nd	14
	Fpr049	28	< 2.5	nd	135	< 2.5	nd	nd	17	< 2.5	nd	nd	< 2.5
	FUS16163	77	19	nd	590	38	6	nd	334	< 2.5	nd	nd	14
5 wpi	Fpr047^b)^	56	10	nd	165	25	3	nd	175	6	nd	nd	30
	FUS049^b)^	51	9	< 2.5	312	4	< 2.5	nd	19	4	nd	nd	28
	FUS16163^b)^	37	8	nd	267	24	6	nd	182	< 2.5	nd	nd	9

*nd*, not detected

^a)^Results of two symptomatic onions

^b)^Results of four symptomatic onions

**Fig. 5:**
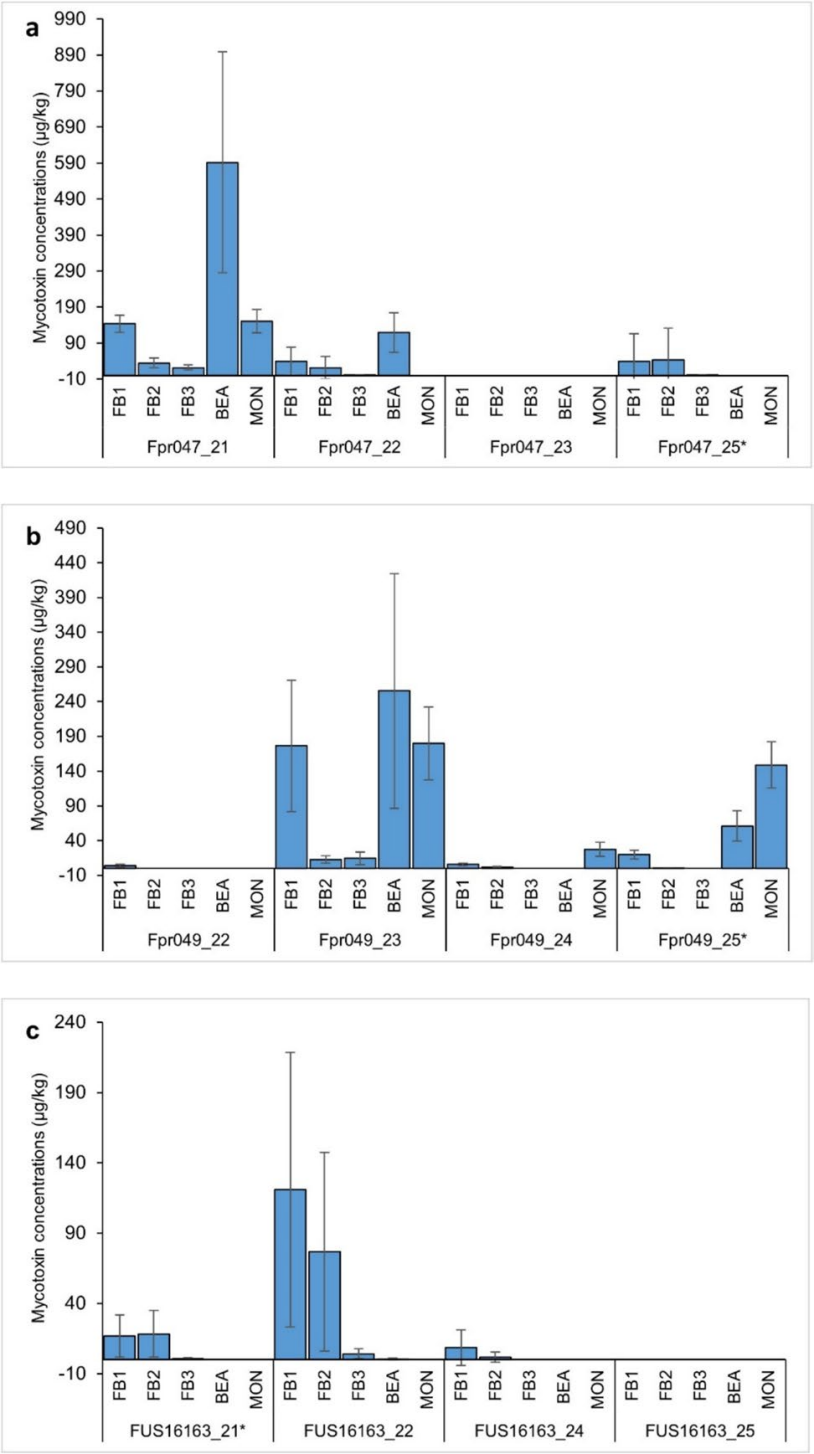
Average concentrations and standard deviations of three fumonisins (FBs), beauvericin (BEA), and moniliformin (MON) at 5 weeks post-inoculation (wpi) in four symptomatic onions inoculated with **a** Fpr047, **b** Fpr049, and **c** FUS16163. Additional onion samples are marked with an asterisk (*). Note that the *Y*-axis scale is different for each isolate

MON was detected at 4 wpi in the symptomatic tissues with Fpr047 and Fpr049 (< 5 µg/kg and 46 ± 26 µg/kg, respectively), but not in the non-symptomatic tissues. In the symptomatic tissue with Fpr047, BEA (10 ± 6.3 µg/kg) was also detected at 4 wpi. Clearly quantifiable concentrations of BEA (≥ 36 µg/kg) and MON (≥ 20 µg/kg) were measured at 5 wpi in the symptomatic tissues with Fpr047 and Fpr049, but not with FUS16163. The average concentrations and standard deviations for all the five mycotoxins at 5 wpi in each onion bulb inoculated with *F. proliferatum* are shown in Fig. 5.

Two of the onions analyzed were assessed as outliers: at 2 wpi in an onion inoculated with Fpr047 (Fpr047_7), MON concentration was about 20 times higher in the non-symptomatic tissue sample (Fpr047_7B; 240 µg/kg) than in the symptomatic tissues (Fpr047_7A; 10 µg/kg). In the other bulbs inoculated with Fpr047 or Fpr049, MON was detected in symptomatic onion tissues at 4 and 5 wpi but was not detected in the non-symptomatic tissues. Another onion sample, inoculated with FUS16163 (FUS16163_12), had high concentrations of FB_1_ (390–4900 µg/kg), FB_2_ (130–1170 µg/kg), and FB_3_ (90–940 µg/kg) in all four symptomatic subsamples at 3 wpi. This sample had to be left as an outlier because also MON (11–24 µg/kg) was detected in all the subsamples, and according to the rice culture analysis, FUS16163 should not be able to produce MON (Table 2).

### Correlation between *FUM1* gene expression and fumonisin concentrations

Correlation between the *FUM1* gene expression level and the amounts of fumonisins produced was analyzed for samples prepared from the same bulbs at 3 and 4 wpi. Even though the different analyses were performed using different subsamples and the amount of fumonisins showed the accumulated products while the detected gene expression level showed the momentary activity, a significant positive correlation was detected between the *FUM1* expression level and fumonisin concentrations. Pearson correlation coefficient was 0.90 and 0.93 for FB_1_ and FB_2_, respectively, and *p*-values < 0.001 for linear regression (Supplementary Data S3). Two samples were discarded as outliers: FUS16163_12A, for which the mycotoxin levels were 1000 fold higher than in the other samples, and Fpr049_20A, for which the *F. proliferatum* gene expression levels were too low to allow quantitation of *FUM1* expression.

## Discussion

This is the first report on a timescale study of mycotoxin gene expression and mycotoxin production by different isolates of *F. proliferatum* during the process of colonizing onion (*A. cepa*) bulbs. Most of the earlier reports on *Fusarium*-infected onions focused on the morphological identification of the fungal species or on the rot and wilting symptoms caused by the fungal isolates, mostly *F. oxysporum* f. sp. *cepae* (Lacy and Roberts 1982; Toit et al. 2003; Ghanbarzadeh et al. 2014; Haapalainen et al. 2016). While *F. oxysporum* f. sp. *cepae* can cause a severe rot symptom in the infected onion tissues and damage the entire bulb (Cramer 2000; Haapalainen et al. 2023), the onions infected with *F. proliferatum* seemed healthy and firm from outside even 5 weeks after inoculation. However, after splitting the bulbs into halves, water-soaked lesions in the scales were observed, growing in upward direction over time and later turning dry and brown. The symptoms were very similar to those of garlic rot caused by *F. proliferatum* in Spain (Gálvez et al. 2017) and the symptoms previously observed at 3 wpi in *A. cepa* inoculated with *F. proliferatum* and *F. oxysporum* f. sp. *cepae* (Haapalainen et al. 2023). After 4 wpi, the symptomatic scales started shrinking and drying, and at 5 wpi, the symptom thus resembled the dry scale disease previously described in onions infected by *F. proliferatum* (Reitz et al. 2016). In this study, we found that for the inoculated onion bulbs, many of the symptomless tissue samples were also highly colonized by *F. proliferatum.* This could be explained by the *Fusarium* fungi being capable of both biotrophic and necrotrophic interaction with the host plant (Lyons et al. 2015).

Detecting gene expression of a fungal pathogen growing inside the host plant can be challenging, due to the relatively small amount of the pathogen in comparison to the host. In this study, we confirmed the expression of the *F. proliferatum* genes *FUM1* and *SIX2-1* within the inoculated onion bulbs. Two homologs of the *SIX2* gene were previously identified in the *F. proliferatum* strain Fpr A8 genomic sequence by Armitage et al. (2019). Haapalainen et al. (2023) found that all the 15 studied *F. proliferatum* isolates from onion harbored *SIX2-1*, and 11 of them also carried the other gene variant *SIX2-2*. In this study, expression of *SIX2-1* was detected in samples of infected onion tissues for all the three *F. proliferatum* isolates. Virulence of the isolate Fpr049, lacking the gene *SIX2-2* (Haapalainen et al. 2023), was comparable to the isolates Fpr047 and FUS16163, suggesting that the function of *SIX2-2* is not essential for the infection of mature onion bulbs.

*FUM1* gene expression was detected for all three *F. proliferatum* isolates within the infected onion tissues at the time points 2, 3, and 4 wpi, and fumonisins were detected in the symptomatic onion tissues at 3, 4, and 5 wpi. Previously, *FUM1* positive *F. proliferatum* isolates from Welsh onion (*A. fistulosum*) were found capable of producing high concentrations of FB_1_ in rice medium (Dissanayake et al. 2009). All the FB_1_-producing isolates were from wilted Welsh onion plants, whereas both producing and nonproducing isolates were found from the Welsh onion seeds. The 15 *F. proliferatum* isolates from *A. cepa* bulbs studied earlier (Haapalainen et al. 2023) and in this work in Finland all harbored the gene *FUM1*, even though some were from diseased and the others from symptomless bulbs. The three *F. proliferatum* isolates studied in this work originated from symptomless onions, one from a mature bulb and two from symptomless sets. This raises the question of the role of fumonisins in the pathogenicity of *F. proliferatum* on Welsh onion and common onion. In this study, *F. proliferatum FUM1* gene expression and onion symptom development showed a significant positive correlation (Fig. 4), suggesting that fumonisins could play a role in the colonization of onion tissues and/or in symptom development. For *Fusarium verticillioides*, it was previously shown that the *FUM* gene cluster is required for pathogenesis on maize seedlings (Glenn et al. 2008), and that FB_1_ facilitates the fungal colonization of maize embryos (Sánchez-Rangel et al. 2012). FB_1_ treatment was also shown to increase the aggressiveness of *F. proliferatum* on banana fruit and to accelerate plant cell death (Xie et al. 2021). The inhibition of plant ceramide synthase by FB_1_ can induce programmed cell death that results in tissue necrosis (Iqbal et al. 2021).

In this study, fumonisin production by three *F. proliferatum* isolates during colonization of onion bulbs was shown to have started before 3 wpi (Table 4). Fpr047 seemed to be a weaker fumonisin producer than FUS16163 and Fpr049 in onion, in contrast to the rice culture where FUS16163 appeared as the weakest fumonisin producer. A large variation was detected in the fumonisin concentrations between the replicate onion tissue samples at 3 and 4 wpi. Typically, no fumonisins were detected in one of the samples, while another sample contained fumonisins either at a trace or quantitative level, and the third one had a high level of fumonisins. This variation could be explained by an uneven distribution of the fungus inside the inoculated bulbs at the early stages of colonization. This is supported by our observation that later, in the 5 wpi samples, the distribution of fumonisins inside the symptomatic area was more homogenous than in the 3 and 4 wpi samples.

Another possible reason for the observed variation in the fumonisin concentrations between the replicate samples at 3 and 4 wpi could be a variation in the expression levels of the mycotoxin-related genes, as a significant positive correlation was found between the *FUM1* gene expression level and both the amount of fumonisins and the size of the visible symptom. In a study of Fanelli et al. (2012), the fumonisin production in *F. proliferatum* was found to be several folds increased under exposure to light, in comparison to dark incubation. In this study, the inoculated onion bulbs were incubated in dark, which could have suppressed the fumonisin production. On the other hand, incubation at temperatures above 20 °C could have enhanced the fumonisin production, since *F. proliferatum* was previously shown to produce more fumonisins at 25 °C than at 15 °C (Cendoya et al. 2017).

Production of BEA and MON by *F. proliferatum* in onion appeared to have started later than the fumonisin production, between 3 and 4 wpi. Fpr049 produced more MON than Fpr047, whereas Fpr047 produced more BEA than Fpr049. FUS16163 did not produce MON in onion, and it was uncertain whether it produced BEA. The results on BEA and MON production in onion (Fig. 5) agree well with the results obtained in the rice culture experiment (Table 2).

In this study, mycotoxin accumulation was detected in the majority of the symptomatic onion tissue samples at 5 wpi. FB_1_ was even detected in a non-symptomatic tissue sample at 5 wpi, from a bulb inoculated with Fpr049, demonstrating that even the symptomless parts of an infected bulb can contain fumonisins. According to Mondani et al. (2021), fumonisins were detected in both the symptomatic and non-symptomatic garlic cloves infected by *F. proliferatum*, after 6 months of storage. In our previous study (Rämö et al. 2021), we detected BEA and MON in harvested onion bulbs naturally colonized by *F. oxysporum* and FB_1_ in the bulbs naturally colonized by *F. proliferatum*. These results suggest a potential risk of *Fusarium*-infected and mycotoxin-containing bulbs getting into fresh and processed products and underline the importance of quality control both after harvest and during cold storage of onion and garlic. However, maximum levels have not been set for fumonisins in onions or other vegetables or fruits. The maximum levels allowed for FB_1_ and FB_2_ have only been set in maize for human consumption in EC-European Commision (2023). The maximum level for FB_1_ + FB_2_, for example, in unprocessed maize grains is 4000 µg/kg, in milled maize products for consumers 1000 µg/kg, and in maize-based baby food 200 µg/kg. Some recommendations are also given for cereal-based animal feed in EC-European Commission (2016). The guidance values for FB_1_ + FB_2_ are higher for animal feed, for example in compound feed for pigs, horses, rabbits, and other pets 5 mg/kg and for poultry 20 mg/kg. Based on this work and the previous studies detecting fumonisins in asparagus and garlic (Seefelder et al. 2002; Mondani et al. 2021), sweet pepper (Monbaliu et al. 2010), pineapple (Stępień et al. 2013), and onion (Rämö et al. 2021), we suggest that maximum-level assessment of fumonisins should be expanded to other food commodities than maize and cereal products.

## Supplementary Information

Below is the link to the electronic supplementary material.

Supplementary file1 (PDF 208 KB)

Supplementary file2 (PDF 27 KB)

Supplementary file3 (PDF 27 KB)

## Data Availability

The IGS DNA sequences of the Fusarium proliferatum isolates Fpr047, Fpr049, and FUS16163 are avaliable in GenBank at www.nlm.nih.gov with accessionnumbers PQ352462, PQ3524463,PQ352464. Accession numbers of TEF sequences of *F. proliferatum* isolates published previously, are presented in the Supplementary Data S1.

## Abbreviations

BEA: Beauvericin
FB_1_: Fumonisin B_1_
FB_2_: Fumonisin B_2_
FB_3_: Fumonisin B_3_
FBR: Fusarium basal rot
Fpr: *Fusarium proliferatum*
IGS: Intergenic spacer
max: Maximum
min: Minimum
MON: Moniliformin
RT: Room temperature
wpi: Week(s) post-inoculation
