# Supplementary material for: Fumonisin production and symptom development in onion (Allium cepa) inoculated with Fusarium proliferatum
Source: Mycotoxin Res. 2025 Jun 14;41(3):457–73. doi: 10.1007/s12550-025-00595-0 (PMC12307559; doi:10.1007/s12550-025-00595-0)
Supplement: Supplementary file 1 — Supplementary file1 (PDF 208 KB) [file 12550_2025_595_MOESM1_ESM.pdf]

Supplementary Data S1 for

Fumonisin production and symptom development in onion (*Allium cepa*) inoculated with *Fusarium proliferatum*

Mycotoxin Research

Sari Rämö, Sadikshya Ghimire, Minna Haapalainen, Satu Latvala

Corresponding author: Sari Rämö, Natural Resources Institute Finland (Luke), Finland, sari.ramo@luke.fi

Supplementary Data S1. Onions inoculated with three *Fusarium proliferatum* (Fpr) isolates (Fpr047, Fpr049 and Fpr919 = FUS16163), and water (H_20) as the control, were placed upside-down on a cardboard support, so that they were not in contact with each other, and kept in the dark at 22 °C until sampling

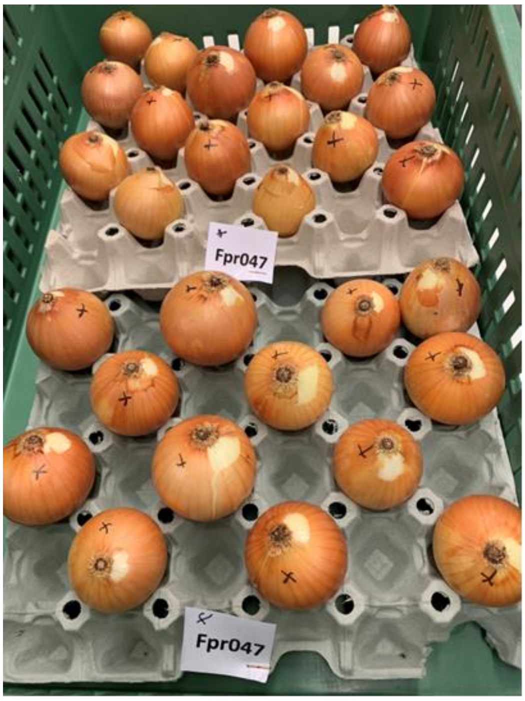
Fpr047
Fpr047

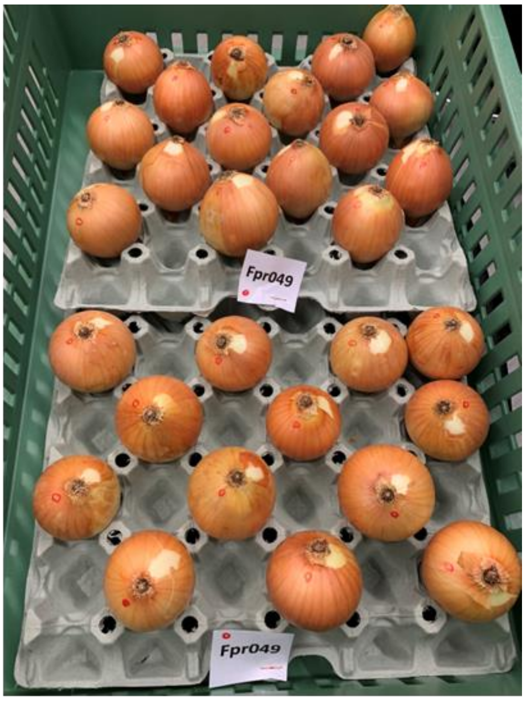
Fpr049
Fpr049

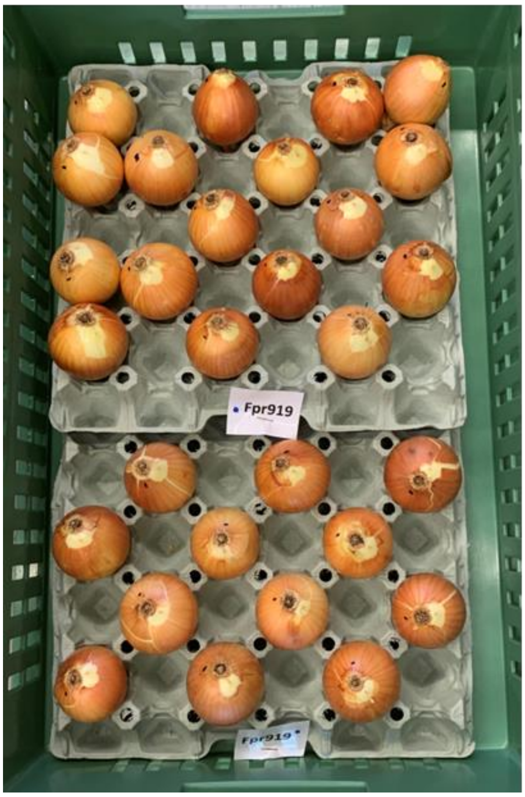
Fpr919
Fpr919

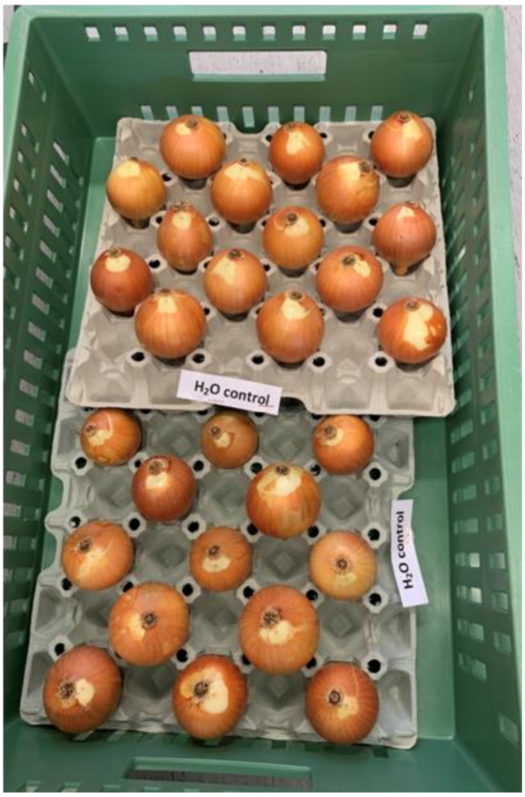
H_2O control
H_2O control
