# Supplementary material for: Fumonisin production and symptom development in onion (Allium cepa) inoculated with Fusarium proliferatum
Source: Mycotoxin Res. 2025 Jun 14;41(3):457–73. doi: 10.1007/s12550-025-00595-0 (PMC12307559; doi:10.1007/s12550-025-00595-0)
Supplement: Supplementary file 2 — Supplementary file2 (PDF 27 KB) [file 12550_2025_595_MOESM2_ESM.pdf]

Supplementary Data S2 for

Fumonisin production and symptom development in onion (*Allium cepa*) inoculated with *Fusarium proliferatum*

Mycotoxin Research

Sari Rämö, Sadikshya Ghimire, Minna Haapalainen, Satu Latvala

Corresponding author: Sari Rämö, Natural Resources Institute Finland (Luke), Finland, sari.ramo@luke.fi

Supplementary Data S2. Comparison of the expression levels of *F. proliferatum* genes *FUM1* and *SIX2-1* within the infected onion bulbs, in samples taken from the symptomatic tissue

Gene Expression	N	n	Mean	SD	SE Mean	Significance
FUM1	27	20	0.24	0.32	0.07	a
SIX2-1	27	12	0.04	0.04	0.01	b

Note: N = total number of samples, n= the number of samples with gene expression expressed, and the different letters in significance column indicate that the mean gene expression values are significantly different ($p<0.05$) for *FUM1* and *SIX2-1*.
