# Supplementary material for: Fumonisin production and symptom development in onion (Allium cepa) inoculated with Fusarium proliferatum
Source: Mycotoxin Res. 2025 Jun 14;41(3):457–73. doi: 10.1007/s12550-025-00595-0 (PMC12307559; doi:10.1007/s12550-025-00595-0)

Supplementary Data S3 for

Fumonisin production and symptom development in onion (*Allium cepa*) inoculated with *Fusarium proliferatum*

Mycotoxin Research

Sari Rämö, Sadikshya Ghimire, Minna Haapalainen, Satu Latvala

Corresponding author: Sari Rämö, Natural Resources Institute Finland (Luke), sari.ramo@luke.fi

Supplementary Data S3. A significant positive correlation was detected between the expression level of *FUM1* and the concentrations of FB_1 and FB_2 toxins, measured from samples of symptomatic onion tissues taken at 3 and 4 weeks post-inoculation. Data on 16 onion bulbs was included in the analysis, and the Pearson correlation coefficient (r) and p-value were calculated for linear regression

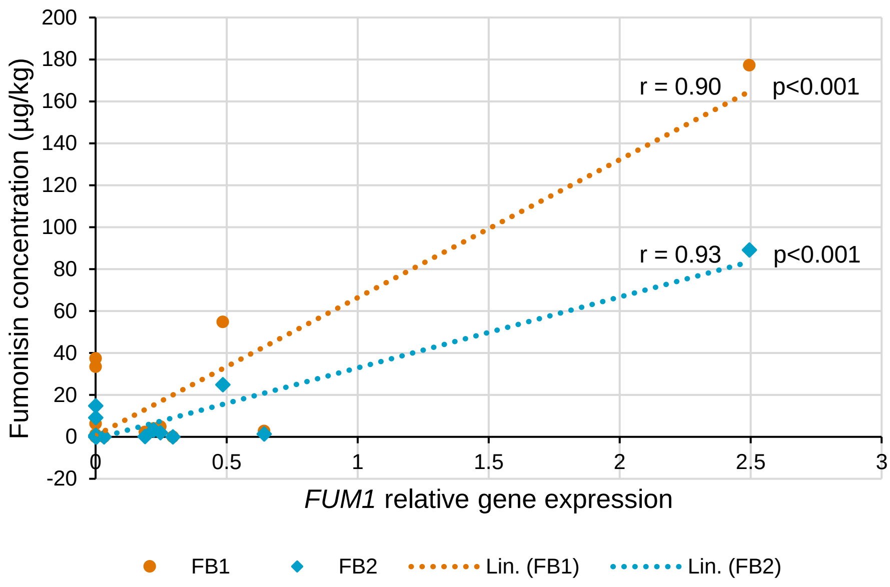

Supplement: Supplementary file 3 — Supplementary file3 (PDF 27 KB) [file 12550_2025_595_MOESM3_ESM.pdf]
